# Salivary biomarkers for the molecular diagnosis of Dementia with Lewy Bodies

**DOI:** 10.1002/alz.092204

**Published:** 2025-01-09

**Authors:** Fabrizia D'Antonio, Giorgio Vivacqua, Marco Serrentino, Martyna Podgajna, Aleksandra Kaczynska, Martina Peconi, Maria Ilenia De Bartolo, Massimiliano Panigutti, Micaela Sepe‐Monti, Giuseppina Talarico, Giovanni Fabbrini, Giuseppe Bruno

**Affiliations:** ^1^ Sapienza University of Rome, Roma, Lazio Italy; ^2^ Campus Biomedico University of Roma, Rome Italy; ^3^ Sapienza University of Rome, Rome Italy; ^4^ IRCCS Neuromed, Pozzilli, Isernia Italy; ^5^ Sapienza University of Rome, Rome, Rome Italy; ^6^ Department of Human Neuroscience, Sapienza University of Rome, Rome Italy

## Abstract

**Background:**

Dementia with Lewy bodies (DLB) is the second most common form of neurodegenerative dementia. Although its prevalence, DLB is under‐investigated and frequently misdiagnosed and there are no available biomarkers. The aim of this study was to investigate salivary a‐synuclein (a‐syn) and tau species in DLB patients as potential disease biomarkers and to assess their potential in differential diagnosis with Alzheimer’s Disease (AD) and Parkinson’s Disease (PD).

**Method:**

We measured total‐ and oligomeric a‐syn, total‐tau and S199‐phosphorylated‐tau (pS199‐tau) in the saliva from 21 DLB, 20 AD, 20 PD patients and 20 Healthy subjects (HC), using quantitative enzyme‐linked immunosorbent Assay (ELISA) analyses.

**Result:**

Salivary total a‐syn was higher in DLB compared to PD, whereas all pathological groups had higher concentration of oligomeric a‐syn compared to HC. Salivary total‐tau concentration was higher in all the pathological groups compared to HC, whereas the concentrations did not differ among patients’ groups. Conversely, salivary levels of pS199‐tau were higher in DLB and AD patients in comparison to both HC and PD patients. Both correlation matrix and Principal Component Analysis showed that core clinical DLB features were related to a‐synuclein pathology, while the cognitive decline was associated with salivary levels of pS199‐tau. Receiver operating characteristic analysis reported high diagnostic accuracy for both a‐syn oligomers and pS199‐tau, between DLB and HC and an adequate accuracy between DLB and PD.

**Conclusion:**

Salivary total and oligomeric a‐syn were accurate in differentiating DLB from HC, whereas pS199‐tau might represent a marker of cognitive decline. These findings provide a preliminary evidence that salivary a‐syn and tau species might be promising in identifying DLB patients with potential implications for early diagnosis and disease modifying therapies.

